# Different responses of avian feeding guilds to spatial and environmental factors across an elevation gradient in the central Himalaya

**DOI:** 10.1002/ece3.5040

**Published:** 2019-03-14

**Authors:** Zhifeng Ding, Jianchao Liang, Yiming Hu, Zhixin Zhou, Hongbin Sun, Lina Liu, Haijun Liu, Huijian Hu, Xingfeng Si

**Affiliations:** ^1^ Guangdong Key Laboratory of Animal Conservation and Resource Utilization, Guangdong Public Laboratory of Wild Animal Conservation and Utilization Guangdong Institute of Applied Biological Resources Guangzhou China; ^2^ School of Environmental Science and Engineering Southern University of Science and Technology Shenzhen China; ^3^ Shenzhen Nature Reserve Management Center Shenzhen China; ^4^ Zhejiang Tiantong Forest Ecosystem National Observation and Research Station, School of Ecological and Environmental Sciences East China Normal University Shanghai China

**Keywords:** bird guild, elevational gradient, environmental factors, Himalaya, hump‐shaped pattern, spatial factors

## Abstract

Although elevational patterns of species richness have been well documented, how the drivers of richness gradients vary across ecological guilds has rarely been reported. Here, we examined the effects of spatial factors (area and mid‐domain effect; MDE) and environmental factors, including metrics of climate, productivity, and plant species richness on the richness of breeding birds across different ecological guilds defined by diet and foraging strategy. We surveyed 12 elevation bands at intervals of 300 m between 1,800 and 5,400 m a.s.l using line‐transect methods throughout the wet season in the central Himalaya, China. Multiple regression models and hierarchical partitioning were used to assess the relative importance of spatial and environmental factors on overall bird richness and guild richness (i.e., the richness of species within each guild). Our results showed that richness for all birds and most guilds displayed hump‐shaped elevational trends, which peaked at an elevation of 3,300–3,600 m, although richness of ground‐feeding birds peaked at a higher elevation band (4,200–4,500 m). The Normalized Difference Vegetation Index (NDVI)—an index of primary productivity—and habitat heterogeneity were important factors in explaining overall bird richness as well as that of insectivores and omnivores, with geometric constraints (i.e., the MDE) of secondary importance. Granivore richness was not related to primary production but rather to open habitats (granivores were negatively influenced by habitat heterogeneity), where seeds might be abundant. Our findings provide direct evidence that the richness–environment relationship is often guild‐specific. Taken together, our study highlights the importance of considering how the effects of environmental and spatial factors on patterns of species richness may differ across ecological guilds, potentially leading to a deeper understanding of elevational diversity gradients and their implications for biodiversity conservation.

## 1 INTRODUCTION

Elevational changes in species diversity and composition have long been of interest to ecologists and naturalists (Lomolino, 2001). Dramatic abiotic and biotic changes can occur over short spatial distances along elevation gradients, making them suitable for examining biodiversity drivers (McCain, 2009). Due to the important insights gained by studying elevation gradients, a plethora of studies evaluating elevational diversity gradients have been conducted in recent decades (Sundqvist, Sanders, & Wardle, 2013). Generally, four main diversity patterns along elevation gradients have been reported: decreasing, low plateau, low plateau with a mid‐elevational peak, and mid‐elevational peaks. Among them, mid‐elevation peaks are the most common richness patterns among vertebrates (45%) at the global scale (McCain & Grytnes, 2010). Several drivers have been proposed to explain these patterns, including the current climate, space, evolutionary history, and biotic processes (reviewed in McCain, 2009). However, spatial (e.g., area, mid‐domain effect (MDE)) (Colwell, Rahbek, & Gotelli, 2004; Rahbek, 1997) and environmental drivers (e.g., climatic variables, productivity, and habitat heterogeneity) are cited most frequently (e.g., Nogués‐Bravo, Araújo, Romdal, & Rahbek, 2008; Sanders & Rahbek, 2012; Wu et al., 2013; Pan et al., 2016; Hu et al., 2017; Elsen, Tingley, Kalyanaraman, Ramesh, & Wilcove, 2017). For example, McCain (2009) conducted a meta‐analysis of global elevational patterns of birds, and found both water and temperature variables were important drivers of elevational diversity gradients.

Specifically, several hypotheses that associate with spatial drivers have been proposed, such as the classic species–area relationship (SAR; e.g., Rosenzweig, 1992, 1995) and spatial constraint hypothesis (MDE; e.g., Colwell et al., 2004; Colwell, Rahbek, & Gotelli, 2005). According to the SAR, larger areas tend to support more species as a result of differential speciation and extinction rates that vary with area at regional and global scales (Rosenzweig, 1992, 1995). The MDE indicated that spatial boundaries would result in greater overlaps in species ranges toward the center of an area and thus maximum diversity at the middle elevation of a mountain (Colwell & Lees, 2000). Similarly, both the climate–richness relationship and productivity–richness relationship were proposed to explain the association between richness and the environment (e.g., McCain, 2007, 2009, 2010; McCain & Grytnes 2010). Climatic variables like temperature and/or precipitation could directly influence taxonomic richness through physiological tolerances, and indirectly influence taxonomic richness through food resource availability (McCain, 2009). In addition, productivity, often estimated using the Normalized Difference Vegetation Index (NDVI) (reviewed in Pettorelli et al., 2006) or habitat heterogeneity, metrics of vegetation height and structural complexity, has positive relationships with species richness. The reason is that areas with higher productivity or greater vegetation height and structural complexity could support more individuals within a community and thus more species, and could also increase the availability of critical resources and therefore accommodate more species (MacArthur & MacArthur, 1961; Srivastava & Lawton, 1998).

Birds have always served as an excellent model system for examining biodiversity drivers because they occur in nearly all climatic zones and habitat types worldwide (McCain, 2009), and their spatial distributions are relatively well known. In recent decades, many studies of the elevational patterns of bird species richness have been emerged. However, despite decades of effort, the mechanisms underlying those elevational patterns of diversity remain poorly understood (Quintero & Jetz, 2018). The concept of bird guilds, which refer to groups of birds exploiting the same class of environmental resources in a similar way (Root, 2001), is fundamental in avian ecology (e.g., Rodríguez, Jansson, & Andrén, 2007; Balestrieri et al., 2015; Ding, Feeley, Hu, & Ding, 2015). Different guilds have unique resource requirements and environmental tolerances and can reflect the temporal variations in food supply, vegetative cover, predators, and other factors (e.g., O'Connell, Jackson, & Brooks, 2000; Kissling, Sekercioglu, & Jetz, 2012; Katuwal et al., 2016). Thus, it is not surprising that different guilds tend to have different diversity gradients and are influenced by different environmental factors. For example, in an analysis of mist‐netted birds in the tropical Andes, Terborgh (1977) found that insectivores showed a peak at mid‐elevations, whereas nectarivore richness was nearly independent of elevation, suggesting a causal connection between elevation and richness mediated via resource levels. Thus, separating the overall richness gradient into the gradients of different guilds could better understand the processes in shaping community structure and their underlying mechanisms (Root, 2001). It could also be useful to assess how multiple species collectively respond to changes in environmental resources or ecological conditions (Block, Finch, & Brennan, 1995). For example, Katuwal et al. (2016) found that insectivore and omnivore richness had similar mid‐elevation peaks, whereas herbivore richness increased at higher elevations. Such differences in elevational richness patterns among feeding guilds may be related to food availability and/or evolutionary history. Similar results were also reported in the tropical Andes as insectivore and overall richness had similar elevational trends (Jankowski et al., 2013; Terborgh, 1977). Consequently, to understand the structure of bird communities and their variations among different vegetation types, it is important to use bird guilds to analyze their responses to changing habitats (O'Connell et al., 2000; Wiens & Rotenberry, 1981).

Theory predicts and previous studies have shown that the environmental drivers of richness may vary across guilds and studying this variation may help elucidate the processes underlying species richness (McCain & Grytnes, 2010; McCain, 2009). For instance, the richness of granivorous birds is especially prevalent in the pioneer and early stages of ecological succession (Wiens & Johnston, 1977) and display an array of adaptations that allow them to exploit open and unpredictable habitats (Díaz & Telleria, 1996). Thus, granivores would show a preference for disturbed and open habitats because such habitats provided forest openings with larger seed banks (Chettri, Deb, Sharma, & Jackson, 2005). The NDVI is likely to reflect the abundance of insects, because they depend on plant productivity; hence, the NDVI may provide reliable information on food abundance for insectivores (Pettorelli et al., 2011). Also, omnivores, as a generalist guild, could directly benefit from supplemental food sources and habitat variability (as assessed by the NDVI). Ground‐feeding species prefer relatively sparsely vegetated foraging sites according to previous analyses (e.g., Moorcroft, Whittingham, Bradbury, & Wilson, 2002; Butler & Gillings, 2004; Whittingham & Evans, 2004; Schaub et al., 2010).

Mountain regions have diverse environments, which are characterized by considerable variations in geology, topography, climate, and land cover, offering an ideal condition for exploring variations in species diversity over short spatial distances (Körner, 2007). The Himalaya is the highest mountain range in the world and thus offers exceptional conditions for studying elevation gradients. The region is a global hotspot for bird species and possesses one of the greatest ecological amplitudes in the world (Körner, 2000; Myers, Mittermeier, Mittermeier, Fonseca, & Kent, 2000). Climate change is expected to affect biodiversity globally, but high‐altitude Himalayan ecosystems are expected to be among the most severely affected by climate warming (Shrestha, Gautam, & Bawa, 2012; Xu et al., 2009). Thus, to understand biodiversity patterns and their responses to changing habitats and/or a changing climate, it is particularly important to apply the guild approach in this mountain system, and to further improve habitat management and conservation.

In the Himalaya, although many previous studies have examined the elevational patterns of bird species richness (e.g., Acharya, Sanders, Vijayan, & Chettri, 2011; Bhatt & Joshi, 2011; Paudel & Šipoš, 2014; Pan et al., 2016; Elsen et al., 2017), to date very few studies have reported how guild richness changes along elevation gradients, and whether these responses are guild‐specific. In this study, we explored the elevational richness patterns of bird guilds and assessed the role of spatial factors (area and MDE) and environmental factors (temperature, precipitation, plant richness, habitat heterogeneity, NDVI) in shaping patterns of bird guild richness. Given that different guilds have unique resource requirements and environmental tolerances, and have been found to respond more strongly to specific factors (as seen above), we thus tested the following predictions: (a) total avian richness and the richness of each guild should have hump‐shaped patterns as a result of the intermediate elevations possibly being the transition zones between different vegetation types which could support more species; (b) the richness of granivores and ground‐feeding species should increase with habitat openness, while the richness of insectivores and omnivores should be most strongly associated with NDVI, an index of primary productivity.

## 2 MATERIALS AND METHODS

### 2.1 Study sites

The study sites were located in the Gyirong Valley (28°15′–29°0′N, 85°6′–85°41′E), which is the westernmost and longest canyon of Mt. Qomolangma National Nature Reserve, lying on the southern slope of the central Himalaya, China. Detailed descriptions about this study site can be found in Pan et al. (2016).

### 2.2 Bird surveys

We surveyed birds using line transects (Bibby, Burgess, Hill, & Mustoe, 2000) covering the elevational range of 1,800–5,400 m a. s. l. Birds were not surveyed at extremely low or high elevations due to geopolitical restrictions at the lowest elevation of 1,800 m, and inaccessible cliffs and glaciers above 5,400 m. We divided the elevational gradient into 12 bands, with intervals of 300 m. Within each band, three transects (from 2 to 3 km) were established covering all habitat types. We restricted the overall length of the transects in each band to 7.5 km to avoid biased samples (Rahbek, 2005), with the aim of ensuring equal sampling efforts across the whole gradient. We recorded bird species richness and performed four surveys during the wet seasons (from May to June in 2012, in August in 2012, from September to October in 2012, and from July to August in 2013). Bird surveys were conducted in the early morning (from 30 min after dawn to 11:00, local time) and in the late afternoon (from 15:00 to 30 min before sunset) and were not conducted in inclement weather (rain or strong winds) (Pan et al., 2016). All surveys were conducted by the same well‐trained observers along all transects and across both years (Jingjing Li, Hongfen Cao, and Li Xie). Based on individual‐ and sample‐based rarefaction analyses, our sampling efforts were sufficient to detect the species richness along this elevational gradient (Hu et al. unpublished data).

To reduce the potential biases in survey data that can arise with seasonal, long‐distance migrants (McCain, 2009; Quintero & Jetz, 2018; Wu et al., 2013), we only considered breeding resident birds for subsequent analyses. Information on the migratory status of each species was compiled from the local literature (The Comprehensive Scientific Expedition to Qinghai‐Xizang Plateau, Chinese Academy of Sciences, 1983). Shorebirds and owls were also excluded due to their highly specific habitats and nocturnal behavior, respectively. As a result, we recorded a total of 151 breeding birds (Supporting information Table S1).

### 2.3 Guild classifications

We grouped all bird species according to two feeding guild categories: diet and foraging strata (Ding et al., 2015). Based on their predominant diets in the Gyirong Valley (The Comprehensive Scientific Expedition to Qinghai‐Xizang Plateau, Chinese Academy of Sciences, 1983), species were grouped into four dietary guild categories (carnivores, insectivores, omnivores, or granivores) and five foraging strata (ground, understory, midstory, canopy, or aerial) (Remsen & Robinson, 1990).

In our analyses, we excluded guilds from the analysis if the maximum richness in *any* elevation band was less than three species because of the lower statistical power (Weiher, Clarke, & Keddy, 1998). Thus, we excluded carnivores, understory, midstory, canopy, and aerial guilds (Supporting information Table S2) and used the richness of all birds, granivores, insectivores, omnivores, and ground‐feeding species as response variables in subsequent analyses, respectively.

### 2.4 Spatial factors

*The MDE*: We randomized species ranges within the bounded domain without replacement to obtain predicted mean values and 95% confidence intervals for each band based on 5,000 simulations (RangeModel 5; Colwell & Lees, 2000, Colwell, 2008; http://purl.oclc.org/range model). The randomization kept the observed range extents and occupancies constant (if a species occurs at bands 1, 3, and 4, but not at band 2, its range extent and occupancy is 4 and 3, respectively), under the precondition of no ranges extending beyond domain limits. Species’ ranges are systematically selected without replacement one at a time, then placed independently and at random (Colwell & Lees, 2000).

*Area*: We calculated the area of each elevation band in the Gyirong Valley using a 30‐m digital elevation map (DEM) (International Scientific & Technical Data Mirror Site, Computer Network Information Center (CNIC), Chinese Academy of Sciences (CAS); http://www.gscloud.cn).

### 2.5 Environmental factors

#### 2.5.1 Mean annual precipitation (precipitation) and mean annual temperature (temperature)

Precipitation and temperature values for the Gyirong Valley were obtained from the WorldClim database (http://www.worldclim.org, 1950–2000) with a resolution of 30 arc‐seconds. The value for each elevation band was calculated as the average of all grid cells within it.

#### 2.5.2 Habitat heterogeneity

Land cover type in each 300‐m elevational band of the Gyirong Valley was obtained from the 300‐m GlobCover land cover data from CNIC, CAS (http://www.gscloud.cn/; date of the download: 2015/10/25), while the Shannon diversity index was used to assess habitat heterogeneity for each elevation band (Turner & Gardner, 2015).

#### 2.5.3 NDVI

NDVI data (2011–2014) for the Gyirong Valley were obtained from the Ministry of Environmental Protection of the People's Republic of China (http://www.zhb.gov.cn), and we averaged the 4‐year data for each elevational band using ERDAS IMAGINE 9.2.

#### 2.5.4 Plant species richness

Using quadrat samples (Bhattarai & Vetaas, 2003), we determined the presence or absence of plant species (abundance data were not collected) through three quadrats (20 × 20 m) per band in September, 2015. Plant species were identified according to *Flora Xizangica *(Wu, 1983).

### 2.6 Data analyses

We performed first‐, second‐, and third‐order polynomial regressions to find the shape of the relationship between elevation and overall bird species richness/guild richness based on corrected Akaike information criterion (AICc, Akaike's information criterion adjusted for small samples) values (McCain, 2009; Wu et al., 2013). A Spearman's rank correlation was used to examine the correlations among the six explanatory factors (area, precipitation, temperature, plant richness, habitat heterogeneity, and NDVI).

We conducted multiple regression analyses to further explore the elevational patterns of overall bird richness and guild richness (richness of overall birds and guilds were normally distributed, Supporting information Table S3). We first selected the most parsimonious model with the lowest AICc value from 127 candidate models (i.e., all possible combinations of the seven explanatory variables) (Anderson, Burnham, & White, 1998). Because all models with ΔAICc <2 were competing, we used a model‐averaging method to assess the relative importance of different variables based on the 127 candidate models (Anderson & Burnham, 2002; Johnson & Omland, 2004). The spatial autocorrelation of the regression residuals could affect the credibility of the results; however, in cases with a limited sample size, it is not feasible to apply spatial autoregressive analyses with seven explanatory variables (12 sites, spatially arranged in six pairs) (Brehm, Colwell, & Kluge, 2007; Hu et al., 2017), and thus, no spatial autocorrelation analysis was performed in this study.

In addition, hierarchical partitioning (Chevan & Sutherland, 1991) was used to identify the explanatory variables that best accounted for the variation in richness of all birds and each guild. This method calculates contributions of each predictor to the total explained variance of a regression model, reducing collinearity problems due to covariance between predictors (Mac Nally 2000; Mac Nally 2002), and has commonly been used to identify the most likely causal factors (Cisneros, Fagan, & Willig, 2015; Olea, Mateo‐Tomás, & Frutos, 2010; Pinkert, Brandl, & Zeuss, 2017). Furthermore, to reduce collinearity among the variables (Table 1), we selected those with high explanatory power but low variance inflation factor (VIF) values; the VIF value of each explanatory variable was <10 (Dormann et al., 2013). We also performed a hierarchical partitioning analysis to determine the relative importance of the selected variables (see Supporting information Table S4 for the selected variables for each guilds): The hierarchical results were generally similar to those for the seven variables used in the above analysis and are presented in Supporting information Figure S1.

**Table 1:** Spearman correlation matrix for the six explanatory factors of bird communities in the Gyirong Valley

	MAP	PSR	HH	MAT	NDVI
Area	−0.993^a^	−0.811^a^	0.014	−0.993^a^	−0.993^a^
MAP		0.783^a^	0.000	0.986^a^	1.000^a^
PSR			0.070	0.832^a^	0.783^a^
HH				−0.021	0.000
MAT					0.986^a^

Note

HH: habitat heterogeneity; MAP: mean annual precipitation; MAT: mean annual temperature; NDVI: the Normalized Difference Vegetation Index; PSR: plant species richness.

a *p* < 0.01.

For all analyses, area and precipitation were log‐transformed to improve normality. All calculations and analyses were performed in R (ver. 3.2.3; R Development Core Team 2015), PAST (ver. 3.0; Hammer, Harper, & Ryan, 2001; http://folk.uio.no/ohammer/past/) and SAM (ver. 4.0; Rangel, Diniz‐Filho, & Bini, 2010; http://www. ecoevol.ufg.br/sam) software.

## 3 RESULTS

### 3.1 Elevational trends in environmental variables and guild richness

**Figure 1:**
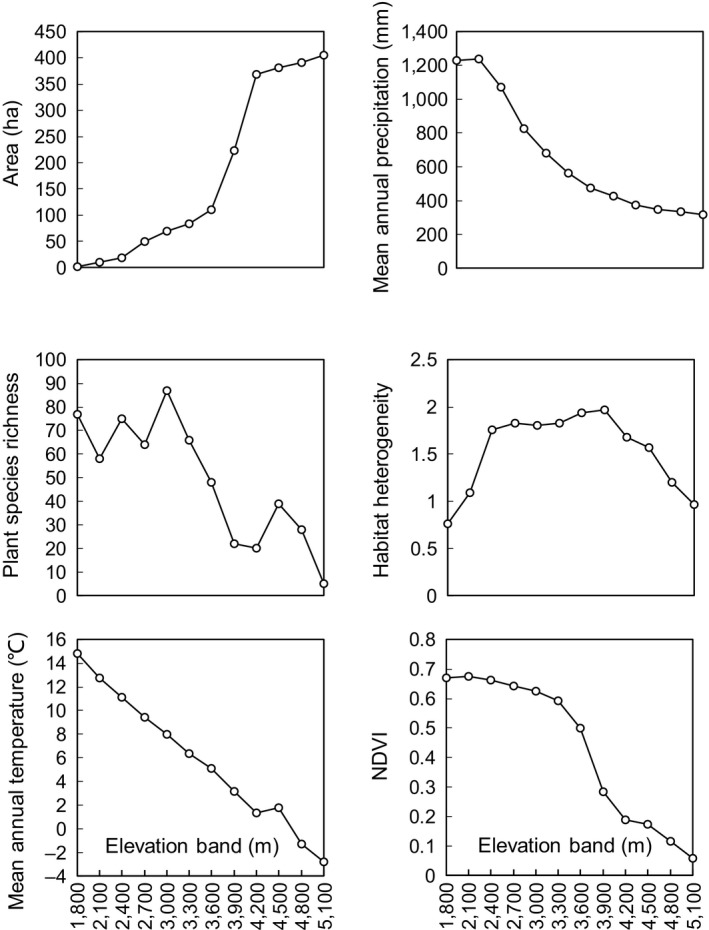
Elevational trends of the six explanatory factors of bird communities in the Gyirong Valley of the central Himalaya

Temperature and precipitation decreased with elevation, whereas area increased monotonically with elevation and showed different trends in comparison with other research (i.e., monotonically decreasing or hump‐shaped patterns). Habitat heterogeneity and plant richness displayed an approximate hump‐shaped pattern along the elevation gradient (Figure 1).

**Figure 2:**
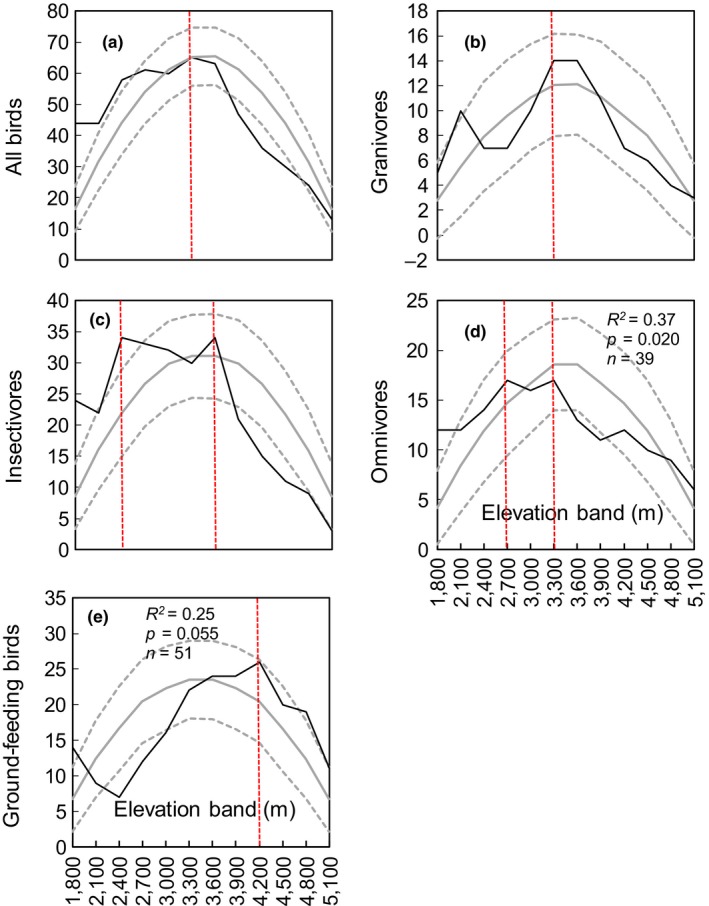
Patterns of overall bird and guild richness along an elevation gradient in the central Himalaya

Generally, overall bird richness and guild richness displayed hump‐shaped elevational trends, but their richness peaks differed (Figure 2). Specifically, overall bird richness increased with elevation up to the sixth elevation band (3,300–3,600 m), and then steadily decreased. Granivore richness had two peaks: a minor peak at the second elevation band (2,100–2,400 m) and another peak at the sixth and seventh bands (3,300–3,600 m and 3,600–3,900 m). Insectivore and omnivore richness also had two peaks, at the third and seventh bands (2,400–2,700 m and 3,600–3,900 m), and at the fourth (2,700–3,000 m) and sixth bands (3,300–3,600 m), respectively. Ground‐feeding species richness peaked at the ninth elevation band (4,200–4,500 m).

The polynomial regressions of the overall bird richness and guild richness patterns indicated that, in general, all bird guilds displayed a hump‐shaped pattern that was better fitted by a quadratic or cubic function rather than by a simple linear regression (Supporting information Table S5).

### 3.2 Relationships between explanatory factors and guild richness

**Figure 3:**
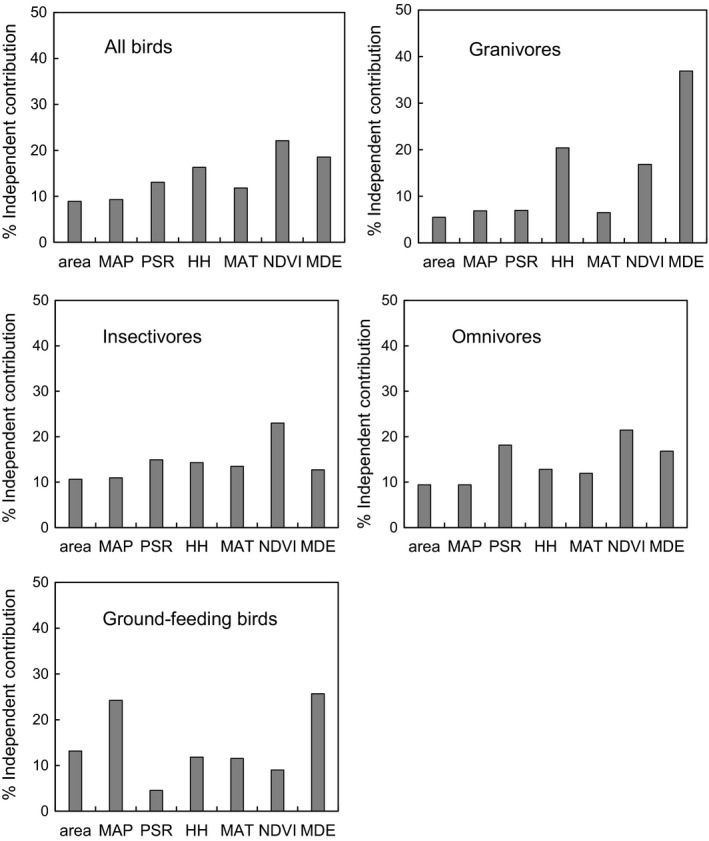
The independent contribution (%) of each variable derived by hierarchical partitioning of overall bird and guild richness

Overall avian species richness was best predicted by the NDVI (positive), precipitation (negative), habitat heterogeneity (positive), and area (negative). However, the importance of environmental and spatial predictors varied across individual guilds. For example, granivore richness was best predicted by the MDE (positive) and habitat heterogeneity (negative). In contrast, insectivore richness was best predicted by the NDVI (positive) and habitat heterogeneity (positive), and omnivore richness was best predicted by the NDVI (positive) and area (positive). Finally, the richness of ground‐feeding birds was best predicted by area (negative), MDE (positive), precipitation (negative), NDVI (negative), and habitat heterogeneity (negative) (Table 2). The model‐averaging analysis produced a similar result as multiple regression analyses (Supporting information Table S6). In general, NDVI was the most important factor influencing overall avian species richness (the independent contribution was 22.08%), insectivores (23.01%), and omnivores (21.47%), whereas MDE had important effects on the richness of granivores (36.90%) and ground‐feeding birds (25.68%), respectively. In addition, habitat heterogeneity also affected the richness of granivores (20.40%) and precipitation also had important effects on the richness of ground‐feeding birds (24.24%) (Figure 3).

**Table 2:** Parameter estimates for the best‐fitted model based on multiple linear regression analyses

Explanatory variables	Coefficient	Standard coefficient	Standard error	*t* statistic	*P*_coeff._	$R_{\text{adj}}^{2}$	*F *statistic	*P*_model_	*AICc wi*
All birds									
Area	−5.003	−0.494	1.602	−3.124	0.02	0.99	242.69	<0.001	0.372
MAP	−27	−0.822	4.994	−5.407	0.002				
HH	21.307	0.522	2.688	7.928	<0.001				
NDVI	74.105	1.078	8.816	8.406	<0.001				
Granivores									
HH	−10.643	−1.213	4.671	−2.279	0.052	0.75	15.23	0.001	0.194
MDE	2.112	1.958	0.574	3.678	0.006				
Insectivores									
HH	11.586	0.446	1.828	6.338	<0.001	0.95	98.41	<0.001	0.652
NDVI	35.482	0.811	3.079	11.524	<0.001				
Omnivores									
Area	1.875	0.953	0.435	4.309	0.003	0.86	31.64	<0.001	0.199
NDVI	21.27	1.593	2.952	7.206	<0.001				
Ground‐feeding birds									
Area	−8.407	−2.191	0.535	12.72	<0.001	0.99	250.16	<0.001	0.999
MDE	2.232	2.157	0.129	17.331	<0.001				
MAP	−16.885	−1.356	1.884	−8.963	<0.001				
HH	−14.2	−0.919	1.737	−8.176	<0.001				
NDVI	−34.937	−1.342	3.55	−9.843	<0.001				

Note

For each species group (all birds, granivores, insectivores, omnivores, and ground‐feeding birds), the best model was selected from the 127 models obtained by forming all possible combinations of seven variables (Area, MAT, MAP, NDVI, HH, MDE, and PSR), guided by the lowest corrected Akaike information criterion value (AICc). Then, we performed multiple regression analysis to obtain parameter estimates for the best‐fitted models based on multiple linear regression analyses. MAP, mean annual precipitation; HH, habitat heterogeneity; NDVI, the Normalized Difference Vegetation Index; and MDE, the mid‐domain effect.

AICc: *wi* refers to the Akaike information criterion weights; *P*_coeff_.: indicates *p*‐values for testing regression coefficients; *P*_model:_ indicates *p*‐values for testing the best‐fitted model.

## 4 DISCUSSION

In this study, we analyzed the spatial and environmental factors determining patterns of bird species richness across a major elevation gradient in the central Himalaya. To do this, we deconstructed the richness gradient by different avian feeding guilds and assessed the guild‐specific drivers of diversity. In general, we found a congruent hump‐shaped pattern of the diversity gradient, but divergent drivers of richness across bird guilds.

### 4.1 Elevational trends in variables and guild richness

The species richness of all breeding birds and guilds in the central Himalaya displayed a hump‐shaped pattern, which support our first prediction. This pattern was the most commonly reported (~45% of cases) in vertebrates (McCain & Grytnes, 2010). Specifically, the richness of all breeding birds and most guilds peaked in the sixth elevation band (3,300–3,600 m), whereas for ground‐feeding species it peaked in the ninth elevation band (4,200–4,500 m); thus, most guilds showed strong congruence with overall bird richness. Similar findings were also reported for an Andes–Amazon gradient: Terborgh (1977) and Jankowski et al. (2013) both found insectivore richness had strong congruence with overall bird richness (mid‐elevation richness peak or decreasing) because insectivores constituted the most species‐rich feeding guild.

In general, diversity peaks at intermediate elevations appear to correspond closely to transition zones between different vegetation types (Lomolino, 2001). In our study, the transition zone between the evergreen broadleaf forest and broadleaf mixed forest (the third and fourth bands), and the transition zone between the dark coniferous forest and shrub and grass (the sixth and seventh bands), might contribute to the richness peaks seen in these regions (Figure 2). The richness of ground‐feeding species peaked at higher elevations versus other guilds. The possible explanation is that shrub and grass occurred at higher elevations (3,900–4,700 m; from the eighth to the tenth band), which is the preferred habitat (i.e., sparsely vegetated foraging sites) for ground‐feeding birds, such as quails and pheasants that are rare and endangered species in China. Compared with mid‐elevation areas, these high‐elevation areas often receive less attention in terms of conservation. In other words, more conservation effort is needed in the high‐elevation areas to protect the habitats used by these endangered species.

It is predicted that temperature decreased monotonically with elevation, whereas plant species richness and habitat heterogeneity have an approximately hump‐shaped pattern (Körner, 2007; McCain & Grytnes, 2010). In this study, variations in temperature, plant species richness, and habitat heterogeneity were consistent with these predicted patterns. However, we found that land area increased monotonically with elevation in the Gyirong Valley, which was contrary to the entrenched idea that land area on a mountain steadily decreases with height (Körner, 2007). This finding indicates we need context‐specific evaluations of the elevation–area relationship of a mountain range during conservation planning (Elsen & Tingley, 2015).

### 4.2 Effects of spatial and environmental factors on guild richness

Given the variation in feeding strategies and their relationship with specific food resources, differences in the elevational distribution of feeding guilds should be expected (Kissling et al., 2012). For example, Hodkinson (2005) found that the availability (and thus the richness) of insects might peak at mid‐elevations, resulting in insectivores showing a peak at mid‐elevations. However, for granivores, Díaz and Telleria (1996) showed that there were stronger associations with open and unpredictable habitats, where plants showed high rates of reproduction and produced large seed crops. In our study, the overall bird richness and guild richness were determined by very different factors (at least partly), which support our second predictions that granivores and ground‐feeding species are more abundant in open habitats, whereas insectivores and omnivores are likely to be associated with NDVI. In addition, we found a positive interaction between habitat heterogeneity and insectivore richness, and a negative interaction between the richness of ground‐feeding birds and NDVI when fitting a single model including guild as an additional term (Supporting information Table S7). These results indicated that guild richness increased with habitat heterogeneity disproportionately for insectivores, and decreased with NDVI disproportionately for ground‐feeding species.

Our finding provided direct evidence that the richness–environment relationship can often be guild‐specific (Kissling et al., 2012). Nevertheless, we found that the NDVI and habitat heterogeneity had a large influence on the richness pattern. This is not surprising because the NDVI has proven extremely useful for predicting species richness and community composition (reviewed in Pettorelli et al., 2011). For example, in East Asia, the average NDVI value was found to be the key factor in determining bird species richness, with a positive linear relationship observed between this value and bird species richness (Ding, Yuan, Geng, Koh, & Lee, 2006; see also Koh, Lee, & Lin, 2006). Associations between the NDVI and bird richness can occur in areas with: (a) high primary productivity (an index of food abundance for birds) (Gordo, 2007), and/or (b) greater vegetation height and structural complexity (i.e., a greater variety of microclimates and microhabitats for a more diverse group of species; Verschuyl, Hansen, McWethy, Sallabanks, & Hutto, 2008). The role of habitat heterogeneity in shaping species richness is often significant (Koh et al., 2006; Rowe, 2009), probably because a wider range of habitat types or greater structural complexity in vegetation can yield more resources and may therefore support a larger number of species (MacArthur & MacArthur, 1961).

Despite a mid‐elevation peak in diversity, the MDE was found to be of secondary importance with respect to bird species richness. The concept of MDE has been controversial since it was first proposed. Hawkins, Diniz‐Filho, and Weis (2005) argued that the concept of geometric constraints did not have a biologically meaningful theoretical foundation, and Hutter, Guayasamin, and Wiens (2013) found that the mid‐domain hypothesis could not explain regional richness patterns; however, Keith and Connolly (2013) argued that geometric constraints can substantially influence regional richness gradients, but were unlikely to drive gradients in local species richness. In this study, we found support for the MDE in driving the richness of all birds, granivores and ground‐feeding species. This can be explained by the possibility that (a) spatial factors might influence the distribution of species indirectly through their correlation with environmental factors, which act on the species more directly, or (b) the species occurring at the ends of the transect have potential ecological amplitudes exceeding the conditions actually realized along the gradient (Kluge, Kessler, & Dunn, 2006).

It is also worthwhile noting that the determinants of the richness of species within each guild varied among guilds. This has been reported across other elevational diversity gradients. For example, in the tropical Andes, insectivores were most severely affected by structural simplification of the habitat, while frugivores were influenced by complex and unresolved factors, such as the availability of fruit crops and plant productivity (Terborgh, 1977). In this study, granivores were negatively influenced by habitat heterogeneity, whereas the other guilds (i.e., insectivores, omnivores, and ground‐feeding species) were positively influenced by NDVI and/or habitat heterogeneity. Hence, while overall bird richness and guild richness were influenced by primary productivity using the NDVI as a proxy (reviewed in Pettorelli et al., 2006), or by habitat heterogeneity (metrics of vegetation height and structural complexity, MacArthur & MacArthur, 1961), our finding that granivore richness was not related to primary production or habitat heterogeneity, but rather to open habitats, where seeds might be abundant. Similar results were also obtained in a study in the eastern Himalayas, where granivores showed a preference for disturbed and open habitats because such habitats provided forest openings with larger seed banks (Chettri et al., 2005).

### 4.3 Caveats and limitations

In this study, we found the hump‐shaped patterns of elevational diversity gradients are generally congruence across bird guilds that peaked at different elevation bands and were explained by divergent spatial and environmental factors. In practice, however, the same patterns could also be driven by other processes, such as historical imprints, instead of ecological limits to diversity (Wiens, 2011). For example, we can expect similar patterns from a pure historical perspective if granivores are all closely related and their ancestor occurred at relatively high elevations, so they may have insufficient time for them to spread across different elevation zones. However, in our study the granivore guild is composed of several families, including Fringillidae, Columbidae, Phasianidae, and Megalaimidae. Similarly, insectivore, and the omnivore guilds are also composed of multiple avian families (Supporting information Figure S2). This indicates that although historical mechanisms, such as niche conservatism could help explain elevational diversity gradients of Himalayan birds (e.g., Wiens & Graham, 2005; Price et al., 2014), ecological processes still play an important role in shaping gradients of richness in this study, given the multiple families in each guild. Some degree of care is required when interpreting our results, because our line‐transect surveys may underestimate rare, or secretive species living in dense forests due to imperfect detection, and thus affect our observed biodiversity patterns (MacKenzie et al., 2017; Si et al., 2018). In our study, the problem of imperfect detection may be more likely to occur in areas of low and mid‐elevations as dense forests were often found in such areas (Wu, 1983), so that future studies may wish to allocate more survey efforts to these areas, or improve the sampling design to target rare species (Specht et al., 2017). Finally, we might need a finer guild classification in future studies to better reflect species functional roles (Pigot, Trisos, & Tobias, 2016).

## 5 CONCLUSION

Our findings provide direct evidence of the richness–environment relationship, which is often guild‐specific (Kissling et al., 2012). Because different guilds showed a preference for different habitats in our study, it is difficult to provide specific recommendations for bird conservation efforts because conservation benefits one guild at the expense of others, and the entire guild needs to be accommodated in management planning (Chettri et al., 2005). Taken together, our study highlights the importance of considering the effects of environmental and spatial factors on patterns of species richness that may differ across ecological guilds. Our guild‐specific results can thus contribute to a better understanding of the factors driving elevational diversity gradients and provide conservation implications for protecting biodiversity in mountainous areas.

## AUTHOR CONTRIBUTIONS

Zhifeng Ding (Z.D.) and Huijian Hu (H.H.) conceived and designed the experiments. Z.D., Jianchao Liang (J.L.), Yiming Hu (Y.H.), Zhixin Zhou (Z.Z.), and H.H. performed the experiments. Z.D., J.L., Y.H., Z.Z., and Xingfeng Si (X.S.) analyzed the data. Z.D., Haijun Liu (H.L.), and X.S. wrote the paper. Z.D., J.L, Y.H., Z.Z., Hongbin Sun (H.S.), Lina Liu (L.L.), and X.S. prepared figures and/or tables. H.L. and H.H. contributed reagents/materials/analysis tools. All authors reviewed drafts of the paper and agreed on the main conclusions of the manuscript.

## Supporting information

Supinfo

## Data Availability

Data are provided as Supporting Information Table S1.

## References

[ece35040-bib-0001] Acharya, B. K. , Sanders, N. J. , Vijayan, L. , & Chettri, B. (2011). Elevational gradients in bird diversity in the Eastern Himalaya: An evaluation of distribution patterns and their underlying mechanisms. PLoS ONE, 6, e29097 10.1371/journal.pone.0029097 22174953 PMC3236786

[ece35040-bib-0002] Anderson, D. R. , & Burnham, K. P. (2002). Avoiding pitfalls when using information‐theoretic methods. Journal of Wildlife Management, 66, 912–918. 10.2307/3803155

[ece35040-bib-0003] Anderson, D. R. , Burnham, K. P. , & White, G. C. (1998). Comparison of akaike information criterion and consistent akaike information criterion for model selection and statistical inference from capture‐recapture studies. Journal of Applied Statistics, 25, 263–282. 10.1080/02664769823250

[ece35040-bib-0004] Balestrieri, R. , Basile, M. , Posillico, M. , Altea, T. , De Cinti, B. , & Matteucci, G. (2015). A guild‐based approach to assessing the influence of beech forest structure on bird communities. Forest Ecology and Management, 356, 216–223. 10.1016/j.foreco.2015.07.011

[ece35040-bib-0005] Bhatt, D. , & Joshi, K. K. (2011). Bird assemblages in natural and urbanized habitats along elevational gradient in Nainital district (western Himalaya) of Uttarakhand state, India. Current Zoology, 57, 318–329. 10.1093/czoolo/57.3.318

[ece35040-bib-0006] Bhattarai, K. R. , & Vetaas, O. R. (2003). Variation in plant species richness of different life forms along a subtropical elevation gradient in the Himalayas, east Nepal. Global Ecology and Biogeography, 12, 327–340. 10.1046/j.1466-822X.2003.00044.x

[ece35040-bib-0007] Bibby, C. J. , Burgess, N. D. , Hill, D. A. , & Mustoe, S. (2000). Bird Census Techniques, 2nd ed. London: Academic Press.

[ece35040-bib-0008] Block, W. M. , Finch, D. M. , & Brennan, L. A. (1995). Single‐species versus multiple‐species approaches for management In Block W. M., Finch D. M., & Brennan L. A. (Eds.), Ecology and management of Neotropical migratory birds: A review and synthesis of critical issues (pp. 461–476). Oxford, UK: Oxford University Press.

[ece35040-bib-0009] Brehm, G. , Colwell, R. K. , & Kluge, J. (2007). The role of environment and mid‐domain effect on moth species richness along a tropical elevational gradient. Global Ecology and Biogeography, 16, 205–219. 10.1111/j.1466-8238.2006.00281.x

[ece35040-bib-0010] Butler, S. J. , & Gillings, S. (2004). Quantifying the effects of habitat structure on prey detectability and accessibility to farmland birds. Ibis, 146, 123–130. 10.1111/j.1474-919X.2004.00352.x

[ece35040-bib-0011] Chettri, N. , Deb, D. C. , Sharma, E. , & Jackson, R. (2005). The relationship between bird communities and habitat: A study along a trekking corridor in the Sikkim Himalaya. Mountain Research and Development, 25, 235–243. 10.1659/0276-4741(2005)025[0235:TRBBCA]2.0.CO;2

[ece35040-bib-0012] Chevan, A. , & Sutherland, M. (1991). Hierarchical partitioning. American Statistician, 45, 90–96.

[ece35040-bib-0013] Cisneros, L. M. , Fagan, M. E. , & Willig, M. R. (2015). Effects of human‐modified landscapes on taxonomic, functional and phylogenetic dimensions of bat biodiversity. Diversity and Distributions, 21, 523–533. 10.1111/ddi.12277

[ece35040-bib-0014] Colwell, R. K. (2008). RangeModel: Tools for exploring and assessing geometric constraints on species richness (the mid‐domain effect) along transects. Ecography, 31, 4–7. 10.1111/j.2008.0906-7590.05347.x

[ece35040-bib-0015] Colwell, R. K. , & Lees, D. C. (2000). The mid‐domain effect: Geometric constraints on the geography of species richness. Trends in Ecology and Evolution, 15, 70–76. 10.1016/S0169-5347(99)01767-X 10652559

[ece35040-bib-0016] Colwell, R. K. , Rahbek, C. , & Gotelli, N. J. (2004). The mid‐domain effect and species richness patterns: What have we learned so far? American Naturalist, 163, E1–E23. 10.1086/382056 15026983

[ece35040-bib-0017] Colwell, R. K. , Rahbek, C. , & Gotelli, N. J. (2005). The middomain effect: There's a baby in the bathwater. American Naturalist, 166, E149–E154. 10.1086/491689

[ece35040-bib-0018] Díaz, M. , & Telleria, J. L. (1996). Granivorous birds in a stable and isolated open habitat within the Amazonian rainforest. Journal of Tropical Ecology, 12, 419–425. 10.1017/S0266467400009615

[ece35040-bib-0019] Ding, T. S. , Yuan, H. W. , Geng, S. , Koh, C. N. , & Lee, P. F. (2006). Macro‐scale bird species richness patterns of the East Asian mainland and islands: Energy, area and isolation. Journal of Biogeography, 33, 683–693. 10.1111/j.1365-2699.2006.01419.x

[ece35040-bib-0020] Ding, Z. F. , Feeley, K. J. , Hu, H. J. , & Ding, P. (2015). Bird guild loss and its determinants on subtropical land‐bridge islands, China. Avian Research, 6, 10 10.1186/s40657-015-0019-9

[ece35040-bib-0021] Dormann, C. F. , Elith, J. , Bacher, S. , Buchmann, C. , Carl, G. , Carré, G. , … Münkemüller, T. (2013). Collinearity: A review of methods to deal with it and a simulation study evaluating their performance. Ecography, 36, 27–46. 10.1111/j.1600-0587.2012.07348.x

[ece35040-bib-0022] Elsen, P. R. , & Tingley, M. W. (2015). Global mountain topography and the fate of montane species under climate change. Nature Climate Change, 5, 772–776. 10.1038/nclimate2656

[ece35040-bib-0023] Elsen, P. R. , Tingley, M. W. , Kalyanaraman, R. , Ramesh, K. , & Wilcove, D. S. (2017). The role of competition, ecotones, and temperature in the elevational distribution of Himalayan birds. Ecology, 98, 337–348. 10.1002/ecy.1669 27869987

[ece35040-bib-0024] The Comprehensive Scientific Expedition to Qinghai‐Xizang Plateau, Chinese Academy of Sciences . (1983). The Avifauna of Xizang. Beijing: Science Press.

[ece35040-bib-0025] Gordo, O. (2007). Why are bird migration dates shifting? A review of weather and climate effects on avian migratory phenology. Climate Research, 35, 37–58. 10.3354/cr00713

[ece35040-bib-0026] Hammer, Ø. , Harper, D. A. T. , & Ryan, P. D. (2001). PAST: Paleontological Statistics software package for education and data analysis. Palaeontologia Electronica, 4, 4.

[ece35040-bib-0027] Hawkins, B. A. , Diniz‐Filho, J. A. F. , & Weis, A. E. (2005). The mid‐domain effect and diversity gradients: Is there anything to learn? American Naturalist, 166, E140–E143. 10.1086/491686 16224716

[ece35040-bib-0028] Hodkinson, I. D. (2005). Terrestrial insects along elevation gradients: Species and community responses to altitude. Biological Reviews, 80, 489–513. 10.1017/S1464793105006767 16094810

[ece35040-bib-0029] Hu, Y. M. , Jin, K. , Huang, Z. W. , Ding, Z. F. , Liang, J. C. , Pan, X. Y. , … Jiang, Z. G. (2017). Elevational patterns of non‐volant small mammal species richness in Gyirong Valley, Central Himalaya: Evaluating multiple spatial and environmental drivers. Journal of Biogeography, 44, 2764–2777. 10.1111/jbi.13102

[ece35040-bib-0030] Hutter, C. R. , Guayasamin, J. M. , & Wiens, J. J. (2013). Explaining Andean megadiversity: The evolutionary and ecological causes of glassfrog elevational richness patterns. Ecology Letters, 16, 1135–1144. 10.1111/ele.12148 23802805

[ece35040-bib-0031] Jankowski, J. E. , Merkord, C. L. , Rios, W. F. , Cabrera, K. G. , Revilla, N. S. , & Silman, M. R. (2013). The relationship of tropical bird communities to tree species composition and vegetation structure along an Andean elevational gradient. Journal of Biogeography, 40, 950–962. 10.1111/jbi.12041

[ece35040-bib-0032] Johnson, J. B. , & Omland, K. S. (2004). Model selection in ecology and evolution. Trends in Ecology and Evolution, 19, 101–108. 10.1016/j.tree.2003.10.013 16701236

[ece35040-bib-0033] Katuwal, H. B. , Basnet, K. , Khanal, B. , Devkota, S. , Rai, S. K. , Gajurel, J. P. , … Nobis, M. P. (2016). Seasonal Changes in Bird Species and Feeding Guilds along Elevational Gradients of the Central Himalayas, Nepal. Plos ONE, 11, e0158362 10.1371/journal.pone.0158362 27367903 PMC4930183

[ece35040-bib-0034] Keith, S. A. , & Connolly, S. R. (2013). Effects of diversity‐dependent colonization–extinction dynamics on the mid‐domain effect. Global Ecology and Biogeography, 22, 773–783. 10.1111/geb.12035

[ece35040-bib-0035] Kissling, W. D. , Sekercioglu, C. H. , & Jetz, W. (2012). Bird dietary guild richness across latitudes, environments and biogeographic regions. Global Ecology and Biogeography, 21, 328–340. 10.1111/j.1466-8238.2011.00679.x

[ece35040-bib-0036] Kluge, J. , Kessler, M. , & Dunn, R. R. (2006). What drives elevational patterns of diversity? A test of geometric constraints, climate and species pool effects for pteridophytes on an elevational gradient in Costa Rica. Global Ecology and Biogeography, 15, 358–371. 10.1111/j.1466-822X.2006.00223.x

[ece35040-bib-0037] Koh, C. N. , Lee, P. F. , & Lin, R. S. (2006). Bird species richness patterns of northern Taiwan: Primary productivity, human population density, and habitat heterogeneity. Diversity and Distributions, 12, 546–554. 10.1111/j.1366-9516.2006.00238.x

[ece35040-bib-0038] Körner, C. (2000). Why are there global gradients in species richness? Mountains might hold the answer. Trends in Ecology and Evolution, 15, 513–514. 10.1016/S0169-5347(00)02004-8

[ece35040-bib-0039] Körner, C. (2007). The use of ‘altitude’ in ecological research. Trends in Ecology and Evolution, 22, 569–574. 10.1016/j.tree.2007.09.006 17988759

[ece35040-bib-0040] Lomolino, M. V. (2001). Elevation gradients of species‐density: Historical and prospective views. Global Ecology and Biogeography, 10, 3–4128. 10.1046/j.1466-822x.2001.00229.x

[ece35040-bib-0041] Mac Nally, R. (2000). Regression and model-building in conservation biology, biogeography and ecology: the distinction between – and reconciliation of –‘predictive’ and ‘explanatory’ models. Biodiversity and Conservation, 9, 655–671.

[ece35040-bib-0042] Mac Nally, R. (2002). Multiple regression and inference in ecology and conservation biology: further comments on identifying important predictor variables. Biodiversity and Conservation, 11, 1397–1401.

[ece35040-bib-0043] MacArthur, R. H. , & MacArthur, J. W. (1961). On bird species diversity. Ecology, 42, 594–598. 10.2307/1932254

[ece35040-bib-0044] MacKenzie, D. I. , Nichols, J. D. , Royle, J. A. , Pollock, K. H. , Bailey, L. , & Hines, J. E. (2017). Occupancy Estimation and Modeling: Inferring Patterns and Dynamics of Species Occurrence, 2nd ed New York, NY: Academic Press.

[ece35040-bib-0045] McCain, C. M. , & Grytnes, J. . (2010) Elevational Gradients in Species Richness In McCain C. M. & Grytnes J. (Eds.), Encyclopedia of Life Sciences (ELS). Chichester: John Wiley & Sons Ltd. 10.1002/9780470015902.a0022548

[ece35040-bib-0046] McCain, C. M. (2007). Could temperature and water availability drive elevational diversity? A global case study for bats. Global Ecology and Biogeography, 16, 4116–4128.

[ece35040-bib-0047] McCain, C. M. (2009). Global analysis of bird elevational diversity. Global Ecology and Biogeography, 18, 346–360. 10.1111/j.1466-8238.2008.00443.x

[ece35040-bib-0048] McCain, C. M. (2010). Global analysis of reptile elevational diversity. Global Ecology and Biogeography, 19, 541–553. 10.1111/j.1466-8238.2010.00528.x

[ece35040-bib-0049] Moorcroft, D. , Whittingham, M. J. , Bradbury, R. B. , & Wilson, J. D. (2002). The selection of stubble fields by wintering granivorous birds reflects vegetation cover and food abundance. Journal of Applied Ecology, 39, 535–547. 10.1046/j.1365-2664.2002.00730.x

[ece35040-bib-0050] Myers, N. , Mittermeier, R. A. , Mittermeier, C. G. , Da Fonseca, G. A. , & Kent, J. (2000). Biodiversity hotspots for conservation priorities. Nature, 403, 853–858. 10.1038/35002501 10706275

[ece35040-bib-0051] Nogués‐Bravo, D. , Araújo, M. B. , Romdal, T. , & Rahbek, C. (2008). Scale effects and human impact on the elevational species richness gradients. Nature, 453, 216–219. 10.1038/nature06812 18464741

[ece35040-bib-0052] O'Connell, T. J. , Jackson, L. E. , & Brooks, R. P. (2000). Bird guilds as indicators of ecological conditions in the central Appalachians. Ecological Applications, 10, 1706–1721.

[ece35040-bib-0053] Olea, P. P. , Mateo‐Tomás, P. , & De Frutos, Á. (2010). Estimating and modelling bias of the hierarchical partitioning public‐domain software: Implications in environmental management and conservation. PLoS ONE, 5, e11698 10.1371/journal.pone.0011698 20657734 PMC2908144

[ece35040-bib-0054] Pan, X. Y. , Ding, Z. F. , Hu, Y. M. , Liang, J. C. , Wu, Y. J. , Si, X. F. , … Jin, K. (2016). Elevational pattern of bird species richness and its causes along a central Himalaya gradient, China. PeerJ, 4, e2636 10.7717/peerj.2636 27833806 PMC5101612

[ece35040-bib-0055] Paudel, P. K. , & Šipoš, J. (2014). Conservation status affects elevational gradient in bird diversity in the Himalaya: A new perspective. Global Ecology and Conservation, 2, 338–348. 10.1016/j.gecco.2014.10.012

[ece35040-bib-0056] Pettorelli, N. , Gaillard, J. M. , Mysterud, A. , Duncan, P. , Stenseth, N. C. , Delorme, D. , … Klein, F. (2006). Using a proxy of plant productivity (NDVI) to find key periods for animal performance: The case of roe deer. Oikos, 112, 565–572. 10.1111/j.0030-1299.2006.14447.x

[ece35040-bib-0057] Pettorelli, N. , Ryan, S. , Mueller, T. , Bunnefeld, N. , Jędrzejewska, B. , Lima, M. , & Kausrud, K. (2011). The Normalized Difference Vegetation Index (NDVI): Unforeseen successes in animal ecology. Climate Research, 46, 15–27. 10.3354/cr00936

[ece35040-bib-0058] Pigot, A. L. , Trisos, C. H. , & Tobias, J. A. (2016). Functional traits reveal the expansion and packing of ecological niche space underlying an elevational diversity gradient in passerine birds. Proceedings of the Royal Society B: Biological Sciences, 283, 20152013 10.1098/rspb.2015.2013 PMC4721085 26740616

[ece35040-bib-0059] Pinkert, S. , Brandl, R. , & Zeuss, D. (2017). Colour lightness of dragonfly assemblages across North America and Europe. Ecography, 40, 1110–1117. 10.1111/ecog.02578 PMC6370790 30741964

[ece35040-bib-0060] Price, T. D. , Hooper, D. M. , Buchanan, C. D. , Johansson, U. S. , Tietze, D. T. , Alström, P. , … Martens, J. (2014). Niche filling slows the diversification of Himalayan songbirds. Nature, 509, 222–225. 10.1038/nature13272 24776798

[ece35040-bib-0061] Quintero, I. , & Jetz, W. (2018). Global elevational diversity and diversification of birds. Nature, 555, 246–250. 10.1038/nature25794 29466335

[ece35040-bib-0063] Rahbek, C. (1997). The relationship among area, elevation, and regional species richness in Neotropical birds. The American Naturalist, 149, 875–902. 10.1086/286028 18811253

[ece35040-bib-0064] Rahbek, C. (2005). The role of spatial scale and the perception of large‐scale species‐richness patterns. Ecology Letters, 8, 224–239. 10.1111/j.1461-0248.2004.00701.x

[ece35040-bib-0065] Rangel, T. F. , Diniz‐Filho, J. A. F. , & Bini, L. M. (2010). SAM: A comprehensive application for Spatial Analysis in Macroecology. Ecography, 33, 46–50. 10.1111/j.1600-0587.2009.06299.x

[ece35040-bib-0066] Remsen, J. V. Jr , & Robinson, S. K. (1990). A classification scheme for foraging behavior of birds in terrestrial habitats. Studies in Avian Biology, 13, 144–160.

[ece35040-bib-0067] Rodríguez, A. , Jansson, G. , & Andrén, H. (2007). Composition of an avian guild in spatially structured habitats supports a competition‐colonization tradeoff. Proceedings of the Royal Society B: Biological Sciences, 274, 1403–1411. 10.1098/rspb.2007.0104 PMC2176207 17389222

[ece35040-bib-0068] Root, R. B. (2001). Guilds In Levin S. A. (Ed.), Encyclopedia of biodiversity (pp. 295–302). San Diego: Academic Press.

[ece35040-bib-0069] Rosenzweig, M. L. (1992). Species diversity gradients: We know more and less than we thought. Journal of Mammalogy, 73, 715–730. 10.2307/1382191

[ece35040-bib-0070] Rosenzweig, M. L. (1995). Species diversity in space and time. Cambridge, UK: Cambridge University Press.

[ece35040-bib-0071] Rowe, R. J. (2009). Environmental and geometric drivers of small mammal diversity along elevational gradients in Utah. Ecography, 32, 411–422. 10.1111/j.1600-0587.2008.05538.x

[ece35040-bib-0072] Sanders, N. J. , & Rahbek, C. (2012). The patterns and causes of elevational diversity gradients. Ecography, 35, 4116–3. 10.1111/j.1600-0587.2011.07338.x

[ece35040-bib-0073] Schaub, M. , Martinez, N. , Tagmann‐Ioset, A. , Weisshaupt, N. , Maurer, M. L. , Reichlin, T. S. , … Arlettaz, R. (2010). Patches of bare ground as a staple commodity for declining ground‐foraging insectivorous farmland birds. PLoS ONE, 5, e13115 10.1371/journal.pone.0013115 20949083 PMC2950849

[ece35040-bib-0074] Shrestha, U. B. , Gautam, S. , & Bawa, K. S. (2012). Widespread climate change in the Himalayas and associated changes in local ecosystems. PLoS ONE, 7, e36741 10.1371/journal.pone.0036741 22615804 PMC3352921

[ece35040-bib-0075] Si, X. , Cadotte, M. W. , Zhao, Y. , Zhou, H. , Zeng, D. , Li, J. , … Tingley, M. W. (2018). The importance of accounting for imperfect detection when estimating functional and phylogenetic community structure. Ecology, 99, 2103–2112. 10.1002/ecy.2438 29944742

[ece35040-bib-0076] Specht, H. M. , Reich, H. T. , Iannarilli, F. , Edwards, M. R. , Stapleton, S. P. , Weegman, M. D. , … Arnold, T. W. (2017). Occupancy surveys with conditional replicates: An alternative sampling design for rare species. Methods in Ecology and Evolution, 8, 1725–1734. 10.1111/2041-210X.12842

[ece35040-bib-0077] Srivastava, D. S. , & Lawton, J. H. (1998). Why more productive sites have more species: An experimental test of theory using tree‐hole communities. American Naturalist, 152, 510–529. 10.1086/286187 18811361

[ece35040-bib-0079] Sundqvist, M. K. , Sanders, N. J. , & Wardle, D. A. (2013). Community and ecosystem responses to elevational gradients: Processes, mechanisms, and insights for global change. Annual Review of Ecology, Evolution, and Systematics, 44, 261–280. 10.1146/annurev-ecolsys-110512-135750

[ece35040-bib-0080] Terborgh, J. (1977). Bird species diversity on an Andean elevational gradient. Ecology, 58, 1007–1019. 10.2307/1936921

[ece35040-bib-0081] Turner, M. G. , & Gardner, R. H. (2015). Landscape ecology in theory and practice: Pattern and process, 2nd ed. New York: Springer.

[ece35040-bib-0082] Verschuyl, J. P. , Hansen, A. J. , McWethy, D. B. , Sallabanks, R. , & Hutto, R. L. (2008). Is the effect of forest structure on bird diversity modified by forest productivity. Ecological Applications, 18, 1155–1170. 10.1890/07-0839.1 18686578

[ece35040-bib-0083] Weiher, E. , Clarke, G. P. , & Keddy, P. A. (1998). Community assembly rules, morphological dispersion, and the coexistence of plant species. Oikos, 81, 309–322. 10.2307/3547051

[ece35040-bib-0084] Whittingham, M. J. , & Evans, K. L. (2004). The effects of habitat structure on predation risk of birds in agricultural landscapes. Ibis, 146, 210–220. 10.1111/j.1474-919X.2004.00370.x

[ece35040-bib-0085] Wiens, J. J. (2011). The causes of species richness patterns across space, time, and clades and the role of “ecological limits”. Quarterly Review of Biology, 86, 75–96. 10.1086/659883 21800635

[ece35040-bib-0086] Wiens, J. J. , & Graham, C. H. (2005). Niche conservatism: Integrating evolution, ecology, and conservation biology. Annual Review of Ecology, Evolution, and Systematics, 36, 519–539. 10.1146/annurev.ecolsys.36.102803.095431

[ece35040-bib-0087] Wiens, J. A. , & Johnston, R. F. (1977). Adaptive correlates of granivory in birds In Pinowski J., & Kendeigh S. (Eds.), Granivorous birds in ecosystems (pp. 301–340). Cambridge: Cambridge University Press.

[ece35040-bib-0088] Wiens, J. A. , & Rotenberry, J. T. (1981). Censusing and the evaluation of avian habitat occupancy. Studies in Avian Biology, 6, 522–532.

[ece35040-bib-0089] Wu, Y. J. , Colwell, R. K. , Rahbek, C. , Zhang, C. L. , Quan, Q. , Wang, C. K. , & Lei, F. M. (2013). Explaining the species richness of birds along an elevational gradient in the subtropical Hengduan Mountains. Journal of Biogeography, 40, 2310–2323.

[ece35040-bib-0090] Wu, C. Y. (Ed.) (1983–1987). Flora Xizangica (Vol. 1–5). Beijing: Science Press.

[ece35040-bib-0091] Xu, J. C. , Grumbine, R. E. , Shrestha, A. , Eriksson, M. , Yang, X. F. , Wang, Y. , & Wilkes, A. (2009). The melting Himalayas: Cascading effects of climate change on water, biodiversity, and livelihoods. Conservation Biology, 23, 520–530. 10.1111/j.1523-1739.2009.01237.x 22748090

