# Supplementary material for: Different responses of avian feeding guilds to spatial and environmental factors across an elevation gradient in the central Himalaya
Source: Ecol Evol. 2019 Mar 14;9(7):4116–28. doi: 10.1002/ece3.5040 (PMC6467856; doi:10.1002/ece3.5040)
Supplement: Supplementary file 1 — Supinfo [file ECE3-9-4116-s001.doc]

**Supporting Information**

**Table S1 Elevational band × species presence/absence matrix for breeding birds (excluding summer visitors, shorebirds and owls) in the Gyirong valley, China.**

|  | Dietary categories | Foraging strata | ele1 | ele2 | ele3 | ele4 | ele5 | ele6 | ele7 | ele8 | ele9 | ele10 | ele11 | ele12 |
| --- | --- | --- | --- | --- | --- | --- | --- | --- | --- | --- | --- | --- | --- | --- |
| *Alectoris_chukar* | Granivores | Ground | 0 | 1 | 0 | 0 | 1 | 0 | 0 | 0 | 0 | 0 | 0 | 0 |
| *Tetraogallus_tibetanus* | Granivores | Ground | 0 | 0 | 0 | 0 | 0 | 0 | 0 | 0 | 0 | 0 | 1 | 1 |
| *Lophura_leucomelanos* | Omnivores | Ground | 1 | 1 | 1 | 1 | 1 | 1 | 0 | 0 | 0 | 0 | 0 | 0 |
| *Perdix_hodgsoniae* | Omnivores | Ground | 0 | 0 | 0 | 0 | 0 | 0 | 0 | 0 | 1 | 1 | 1 | 0 |
| *Lophophorus_impejanus* | Granivores | Ground | 0 | 0 | 0 | 0 | 0 | 0 | 1 | 0 | 0 | 0 | 0 | 0 |
| *Ithaginis_cruentus* | Granivores | Ground | 0 | 0 | 0 | 0 | 0 | 1 | 1 | 0 | 0 | 1 | 0 | 0 |
| *Treron_sphenurus* | Granivores | Canopy | 1 | 1 | 1 | 0 | 0 | 0 | 0 | 0 | 0 | 0 | 0 | 0 |
| *Columba_rupestris* | Granivores | Ground | 0 | 0 | 0 | 1 | 1 | 1 | 1 | 1 | 1 | 1 | 1 | 1 |
| *Columba_livia* | Granivores | Ground | 1 | 1 | 1 | 0 | 1 | 0 | 0 | 0 | 0 | 0 | 0 | 0 |
| *Columba_hodgsonii* | Granivores | Canopy | 0 | 1 | 1 | 1 | 1 | 1 | 1 | 1 | 0 | 0 | 0 | 0 |
| *Columba_leuconota* | Granivores | Ground | 0 | 0 | 0 | 0 | 0 | 1 | 1 | 1 | 0 | 0 | 0 | 0 |
| *Streptopelia_orientalis* | Granivores | Ground | 1 | 1 | 1 | 1 | 1 | 1 | 0 | 0 | 0 | 0 | 0 | 0 |
| *Gallinula_chloropus* | Omnivores | Ground | 0 | 0 | 0 | 0 | 0 | 0 | 1 | 0 | 0 | 0 | 0 | 0 |
| *Cuculus_sparverioides* | Insectivores | Canopy | 0 | 0 | 1 | 1 | 0 | 0 | 0 | 0 | 0 | 0 | 0 | 0 |
| *Cuculus_micropterus* | Insectivores | Canopy | 0 | 0 | 1 | 1 | 1 | 0 | 0 | 0 | 0 | 0 | 0 | 0 |
| *Falco_tinnunculus* | Carnivores | Air | 0 | 0 | 0 | 0 | 0 | 0 | 0 | 1 | 1 | 1 | 1 | 0 |
| *Lanius_tephronotus* | Insectivores | Midstorey | 0 | 0 | 1 | 1 | 1 | 1 | 1 | 1 | 0 | 0 | 0 | 0 |
| *Nucifraga_caryocatactes* | Granivores | Canopy | 0 | 1 | 1 | 1 | 1 | 1 | 1 | 0 | 0 | 0 | 0 | 0 |
| *Corvus_corax* | Omnivores | Ground | 0 | 0 | 0 | 0 | 0 | 0 | 0 | 0 | 1 | 0 | 0 | 0 |
| *Corvus_macrorhynchos* | Omnivores | Ground | 1 | 1 | 1 | 1 | 1 | 1 | 1 | 1 | 1 | 1 | 1 | 0 |
| *Corvus_torquatus* | Omnivores | Ground | 0 | 0 | 0 | 0 | 0 | 1 | 0 | 0 | 0 | 0 | 0 | 0 |
| *Urocissa_flavirostris* | Omnivores | Understorey | 1 | 1 | 1 | 1 | 1 | 1 | 0 | 0 | 0 | 0 | 0 | 0 |
| *Cissa_chinensis* | Insectivores | Ground | 0 | 0 | 0 | 1 | 0 | 0 | 0 | 0 | 0 | 0 | 0 | 0 |
| *Pyrrhocorax_pyrrhocorax* | Omnivores | Ground | 0 | 0 | 0 | 1 | 1 | 1 | 1 | 1 | 1 | 1 | 1 | 1 |
| *Pyrrhocorax_graculus* | Omnivores | Ground | 0 | 0 | 0 | 0 | 0 | 0 | 1 | 1 | 1 | 1 | 1 | 1 |
| *Dicrurus_macrocercus* | Insectivores | Midstorey | 0 | 1 | 1 | 0 | 0 | 0 | 0 | 0 | 0 | 0 | 0 | 0 |
| *Oriolus_traillii* | Omnivores | Canopy | 0 | 0 | 0 | 1 | 0 | 0 | 0 | 0 | 0 | 0 | 0 | 0 |
| *Pericrocotus_ethologus* | Insectivores | Canopy | 0 | 1 | 1 | 1 | 0 | 0 | 0 | 0 | 0 | 0 | 0 | 0 |
| *Pericrocotus_brevirostris* | Insectivores | Canopy | 0 | 1 | 1 | 1 | 0 | 0 | 0 | 0 | 0 | 0 | 0 | 0 |
| *Culicicapa_ceylonensis* | Insectivores | Canopy | 1 | 1 | 1 | 0 | 0 | 0 | 0 | 0 | 0 | 0 | 0 | 0 |
| *Chelidorhynx_hypoxantha* | Insectivores | Understorey | 0 | 1 | 1 | 1 | 1 | 1 | 0 | 0 | 0 | 0 | 0 | 0 |
| *Pseudopodoces_humilis* | Insectivores | Ground | 0 | 0 | 0 | 0 | 0 | 0 | 0 | 1 | 1 | 1 | 1 | 0 |
| *Parus_dichrous* | Insectivores | Midstorey | 0 | 0 | 1 | 1 | 1 | 1 | 1 | 0 | 0 | 0 | 0 | 0 |
| *Parus_ater* | Insectivores | Canopy | 0 | 0 | 1 | 1 | 1 | 1 | 1 | 0 | 0 | 0 | 0 | 0 |
| *Parus_rubidiventris* | Omnivores | Canopy | 0 | 0 | 1 | 1 | 1 | 1 | 1 | 1 | 0 | 0 | 0 | 0 |
| *Parus_monticolus* | Insectivores | Canopy | 1 | 1 | 1 | 1 | 1 | 0 | 0 | 0 | 0 | 0 | 0 | 0 |
| *Parus_major* | Insectivores | Canopy | 1 | 0 | 0 | 0 | 0 | 0 | 0 | 0 | 0 | 0 | 0 | 0 |
| *Eremophila_alpestris* | Omnivores | Ground | 0 | 0 | 0 | 0 | 0 | 0 | 0 | 1 | 1 | 1 | 1 | 1 |
| *Calandrella_brachydactyla* | Omnivores | Ground | 0 | 0 | 0 | 0 | 0 | 0 | 0 | 0 | 0 | 0 | 1 | 1 |
| *Alauda_gulgula* | Omnivores | Ground | 0 | 0 | 0 | 0 | 0 | 1 | 1 | 1 | 1 | 1 | 1 | 0 |
| *Hypsipetes_leucocephalus* | Omnivores | Canopy | 1 | 1 | 1 | 0 | 0 | 0 | 0 | 0 | 0 | 0 | 0 | 0 |
| *Pycnonotus_leucogenys* | Omnivores | Canopy | 1 | 1 | 1 | 0 | 0 | 0 | 0 | 0 | 0 | 0 | 0 | 0 |
| *Pycnonotus_jocosus* | Omnivores | Canopy | 1 | 0 | 0 | 0 | 0 | 0 | 0 | 0 | 0 | 0 | 0 | 0 |
| *Garrulax_erythrocephalus* | Omnivores | Understorey | 0 | 0 | 0 | 1 | 0 | 0 | 0 | 0 | 0 | 0 | 0 | 0 |
| *Garrulax_variegatus* | Insectivores | Understorey | 0 | 0 | 1 | 1 | 1 | 1 | 1 | 1 | 0 | 0 | 0 | 0 |
| *Garrulax_affinis* | Omnivores | Understorey | 0 | 1 | 0 | 1 | 0 | 1 | 1 | 1 | 0 | 0 | 0 | 0 |
| *Garrulax_lineatus* | Omnivores | Understorey | 1 | 1 | 1 | 1 | 1 | 0 | 1 | 0 | 0 | 0 | 0 | 0 |
| *Garrulax_ocellatus* | Omnivores | Understorey | 0 | 1 | 1 | 1 | 1 | 1 | 1 | 0 | 0 | 0 | 0 | 0 |
| *Garrulax_chinensis* | Omnivores | Understorey | 0 | 0 | 0 | 0 | 0 | 1 | 0 | 0 | 0 | 0 | 0 | 0 |
| *Minla_strigula* | Omnivores | Canopy | 0 | 1 | 0 | 0 | 1 | 0 | 0 | 0 | 0 | 0 | 0 | 0 |
| *Heterophasia_capistrata* | Omnivores | Midstorey | 1 | 1 | 1 | 1 | 1 | 1 | 0 | 0 | 0 | 0 | 0 | 0 |
| *Yuhina_flavicollis* | Omnivores | Midstorey | 0 | 0 | 0 | 0 | 0 | 0 | 1 | 0 | 0 | 0 | 0 | 0 |
| *Yuhina_gularis* | Omnivores | Midstorey | 0 | 0 | 0 | 1 | 1 | 1 | 0 | 0 | 0 | 0 | 0 | 0 |
| *Yuhina_occipitalis* | Omnivores | Canopy | 0 | 0 | 1 | 1 | 1 | 1 | 0 | 0 | 0 | 0 | 0 | 0 |
| *Pnoepyga_albiventer* | Insectivores | Ground | 0 | 0 | 1 | 0 | 0 | 0 | 0 | 0 | 0 | 0 | 0 | 0 |
| *Alcippe_vinipectus* | Insectivores | Understorey | 0 | 0 | 1 | 1 | 1 | 1 | 1 | 1 | 0 | 0 | 0 | 0 |
| *Prinia_hodgsonii* | Insectivores | Understorey | 1 | 0 | 0 | 0 | 0 | 0 | 0 | 0 | 0 | 0 | 0 | 0 |
| *Prinia_crinigera* | Insectivores | Understorey | 0 | 0 | 1 | 0 | 0 | 0 | 1 | 0 | 0 | 0 | 0 | 0 |
| *Cettia_major* | Insectivores | Understorey | 0 | 0 | 0 | 0 | 0 | 0 | 1 | 0 | 0 | 0 | 0 | 0 |
| *Cettia_brunnifrons* | Insectivores | Understorey | 0 | 0 | 1 | 1 | 1 | 1 | 1 | 0 | 0 | 0 | 0 | 0 |
| *Cettia_pallidipes* | Insectivores | Understorey | 0 | 0 | 0 | 1 | 0 | 0 | 0 | 0 | 0 | 0 | 0 | 0 |
| *Tesia_castaneocoronata* | Omnivores | Understorey | 0 | 0 | 1 | 1 | 1 | 0 | 0 | 0 | 0 | 0 | 0 | 0 |
| *Seicercus_burkii* | Insectivores | Understorey | 0 | 0 | 0 | 1 | 1 | 1 | 0 | 0 | 0 | 0 | 0 | 0 |
| *Phylloscopus_xanthoschistos* | Insectivores | Canopy | 1 | 1 | 1 | 1 | 0 | 0 | 0 | 0 | 0 | 0 | 0 | 0 |
| *Phylloscopus_trochiloides* | Insectivores | Canopy | 1 | 0 | 0 | 0 | 1 | 1 | 1 | 1 | 0 | 0 | 0 | 0 |
| *Phylloscopus_pulcher* | Insectivores | Canopy | 0 | 1 | 0 | 0 | 0 | 0 | 1 | 0 | 0 | 0 | 0 | 0 |
| *Phylloscopus_maculipennis* | Insectivores | Canopy | 0 | 0 | 1 | 0 | 0 | 0 | 0 | 0 | 0 | 0 | 0 | 0 |
| *Phylloscopus_humei* | Insectivores | Canopy | 0 | 0 | 0 | 0 | 0 | 0 | 1 | 0 | 0 | 0 | 0 | 0 |
| *Phylloscopus_chloronotus* | Insectivores | Canopy | 1 | 1 | 1 | 1 | 1 | 1 | 1 | 1 | 0 | 0 | 0 | 0 |
| *Phylloscopus_fuscatus* | Insectivores | Understorey | 0 | 0 | 0 | 0 | 1 | 0 | 0 | 0 | 0 | 0 | 0 | 0 |
| *Phylloscopus_affinis* | Insectivores | Midstorey | 1 | 1 | 1 | 1 | 1 | 1 | 1 | 1 | 1 | 1 | 1 | 0 |
| *Aegithalos_iouschistos* | Insectivores | Canopy | 0 | 0 | 1 | 1 | 1 | 0 | 0 | 0 | 0 | 0 | 0 | 0 |
| *Aegithalos_concinnus* | Insectivores | Canopy | 1 | 1 | 1 | 1 | 0 | 0 | 0 | 0 | 0 | 0 | 0 | 0 |
| *Hirundo_rupestris* | Insectivores | Air | 0 | 0 | 0 | 0 | 1 | 0 | 1 | 1 | 1 | 1 | 0 | 0 |
| *Riparia_riparia* | Insectivores | Air | 0 | 0 | 0 | 0 | 0 | 0 | 0 | 0 | 1 | 0 | 0 | 0 |
| *Cinclus_cinclus* | Insectivores | Ground | 0 | 0 | 0 | 0 | 0 | 0 | 0 | 1 | 1 | 1 | 1 | 0 |
| *Cinclus_pallasii* | Insectivores | Ground | 1 | 0 | 0 | 0 | 0 | 0 | 1 | 0 | 0 | 0 | 0 | 0 |
| *Turdus_viscivorus* | Omnivores | Ground | 0 | 0 | 0 | 0 | 0 | 0 | 1 | 0 | 0 | 0 | 0 | 0 |
| *Tarsiger_chrysaeus* | Insectivores | Understorey | 0 | 0 | 1 | 1 | 0 | 0 | 1 | 0 | 0 | 0 | 0 | 0 |
| *Tarsiger_indicus* | Insectivores | Understorey | 0 | 0 | 0 | 0 | 0 | 0 | 1 | 0 | 0 | 0 | 0 | 0 |
| *Tarsiger_cyanurus* | Insectivores | Understorey | 0 | 0 | 0 | 0 | 1 | 1 | 1 | 1 | 0 | 0 | 0 | 0 |
| *Luscinia_brunnea* | Insectivores | Understorey | 0 | 0 | 0 | 1 | 0 | 0 | 0 | 0 | 0 | 0 | 0 | 0 |
| *Enicurus_scouleri* | Insectivores | Ground | 1 | 0 | 0 | 0 | 0 | 1 | 1 | 0 | 0 | 0 | 0 | 0 |
| *Myophonus_caeruleus* | Insectivores | Ground | 1 | 1 | 1 | 1 | 1 | 1 | 1 | 0 | 0 | 0 | 0 | 0 |
| *Ficedula_superciliaris* | Insectivores | Midstorey | 0 | 0 | 1 | 0 | 0 | 0 | 1 | 0 | 0 | 0 | 0 | 0 |
| *Ficedula_strophiata* | Omnivores | Understorey | 1 | 1 | 0 | 1 | 1 | 1 | 0 | 0 | 0 | 0 | 0 | 0 |
| *Ficedula_tricolor* | Insectivores | Understorey | 0 | 0 | 0 | 1 | 1 | 1 | 0 | 0 | 0 | 0 | 0 | 0 |
| *Phoenicurus_ochruros* | Insectivores | Understorey | 0 | 0 | 0 | 0 | 0 | 0 | 1 | 1 | 1 | 1 | 1 | 1 |
| *Phoenicurus_erythrogastrus* | Insectivores | Ground | 0 | 0 | 0 | 0 | 0 | 1 | 0 | 1 | 1 | 1 | 1 | 0 |
| *Rhyacornis_fuliginosa* | Insectivores | Ground | 1 | 1 | 1 | 1 | 1 | 1 | 1 | 0 | 1 | 0 | 0 | 0 |
| *Chaimarrornis_leucocephalus* | Insectivores | Ground | 1 | 0 | 0 | 0 | 1 | 1 | 1 | 1 | 0 | 0 | 0 | 0 |
| *Phoenicurus_frontalis* | Insectivores | Understorey | 1 | 0 | 0 | 1 | 1 | 1 | 1 | 1 | 0 | 0 | 0 | 0 |
| *Saxicola_torquatus* | Insectivores | Understorey | 0 | 0 | 0 | 0 | 0 | 0 | 1 | 1 | 1 | 1 | 1 | 0 |
| *Saxicola_ferreus* | Insectivores | Midstorey | 0 | 1 | 1 | 1 | 1 | 1 | 0 | 0 | 0 | 0 | 0 | 0 |
| *Oenanthe_deserti* | Insectivores | Ground | 0 | 0 | 0 | 0 | 0 | 0 | 0 | 1 | 0 | 1 | 1 | 1 |
| *Monticola_solitarius* | Insectivores | Ground | 0 | 0 | 0 | 0 | 0 | 0 | 0 | 0 | 1 | 1 | 0 | 0 |
| *Monticola_rufiventris* | Insectivores | Ground | 0 | 0 | 0 | 0 | 0 | 0 | 1 | 0 | 0 | 0 | 0 | 0 |
| *Luscinia_pectoralis* | Insectivores | Understorey | 0 | 0 | 0 | 0 | 0 | 0 | 0 | 0 | 0 | 1 | 0 | 0 |
| *Hodgsonius_phaenicuroides* | Insectivores | Understorey | 0 | 0 | 0 | 0 | 0 | 0 | 1 | 0 | 0 | 0 | 0 | 0 |
| *Niltava_sundara* | Insectivores | Understorey | 0 | 0 | 1 | 1 | 1 | 1 | 1 | 0 | 0 | 0 | 0 | 0 |
| *Eumyias_thalassinus* | Insectivores | Canopy | 1 | 1 | 1 | 0 | 0 | 0 | 0 | 0 | 0 | 0 | 0 | 0 |
| *Muscicapa_sibirica* | Insectivores | Canopy | 1 | 1 | 1 | 0 | 0 | 0 | 0 | 0 | 0 | 0 | 0 | 0 |
| *Troglodytes_troglodytes* | Insectivores | Understorey | 0 | 0 | 0 | 0 | 0 | 1 | 0 | 0 | 1 | 0 | 0 | 0 |
| *Tichodroma_muraria* | Insectivores | Ground | 1 | 0 | 0 | 0 | 1 | 1 | 1 | 0 | 1 | 0 | 0 | 0 |
| *Certhia_familiaris* | Insectivores | Midstorey | 0 | 0 | 1 | 0 | 1 | 1 | 0 | 0 | 0 | 0 | 0 | 0 |
| *Certhia_nipalensis* | Insectivores | Midstorey | 0 | 0 | 0 | 1 | 1 | 0 | 0 | 0 | 0 | 0 | 0 | 0 |
| *Sitta_himalayensis* | Insectivores | Midstorey | 1 | 1 | 1 | 0 | 0 | 0 | 0 | 0 | 0 | 0 | 0 | 0 |
| *Aethopyga_nipalensis* | Omnivores | Canopy | 1 | 0 | 1 | 1 | 1 | 1 | 0 | 0 | 0 | 0 | 0 | 0 |
| *Aethopyga_ignicauda* | Omnivores | Understorey | 0 | 0 | 1 | 0 | 1 | 0 | 0 | 0 | 0 | 0 | 0 | 0 |
| *Dicaeum_ignipectus* | Omnivores | Canopy | 1 | 1 | 1 | 0 | 0 | 0 | 0 | 0 | 0 | 0 | 0 | 0 |
| *Prunella_fulvescens* | Omnivores | Ground | 0 | 0 | 0 | 0 | 0 | 0 | 1 | 1 | 1 | 1 | 1 | 1 |
| *Prunella_rubeculoides* | Omnivores | Ground | 0 | 0 | 0 | 0 | 0 | 0 | 0 | 0 | 1 | 1 | 1 | 1 |
| *Passer_montanus* | Omnivores | Ground | 1 | 0 | 0 | 1 | 0 | 1 | 1 | 1 | 1 | 0 | 0 | 0 |
| *Montifringilla_adamsi* | Omnivores | Ground | 0 | 0 | 0 | 0 | 0 | 0 | 0 | 0 | 1 | 1 | 0 | 0 |
| *Motacilla_alba* | Insectivores | Ground | 1 | 0 | 0 | 0 | 1 | 1 | 1 | 1 | 1 | 0 | 1 | 0 |
| *Motacilla_citreola* | Insectivores | Ground | 0 | 0 | 0 | 0 | 0 | 0 | 0 | 1 | 0 | 0 | 0 | 0 |
| *Motacilla_cinerea* | Insectivores | Ground | 1 | 1 | 0 | 0 | 1 | 1 | 1 | 1 | 0 | 0 | 0 | 0 |
| *Anthus_roseatus* | Omnivores | Ground | 0 | 0 | 0 | 0 | 0 | 0 | 0 | 1 | 1 | 1 | 0 | 0 |
| *Anthus_hodgsoni* | Insectivores | Ground | 1 | 0 | 0 | 1 | 1 | 1 | 1 | 0 | 1 | 0 | 0 | 0 |
| *Mycerobas_carnipes* | Granivores | Canopy | 0 | 0 | 0 | 1 | 1 | 1 | 1 | 1 | 0 | 0 | 0 | 0 |
| *Carduelis_spinoides* | Granivores | Canopy | 1 | 1 | 1 | 1 | 1 | 1 | 0 | 0 | 0 | 0 | 0 | 0 |
| *Serinus_pusillus* | Granivores | Ground | 0 | 0 | 0 | 0 | 0 | 0 | 1 | 0 | 0 | 0 | 0 | 0 |
| *Carduelis_flavirostris* | Granivores | Understorey | 0 | 0 | 0 | 0 | 0 | 0 | 0 | 1 | 1 | 1 | 0 | 0 |
| *Pyrrhula_erythrocephala* | Granivores | Understorey | 0 | 1 | 0 | 0 | 1 | 1 | 1 | 0 | 0 | 0 | 0 | 0 |
| *Haematospiza_sipahi* | Granivores | Canopy | 0 | 0 | 0 | 0 | 0 | 0 | 1 | 0 | 0 | 0 | 0 | 0 |
| *Carpodacus_rodochroa* | Granivores | Understorey | 0 | 0 | 0 | 0 | 0 | 1 | 1 | 0 | 0 | 0 | 0 | 0 |
| *Carpodacus_thura* | Granivores | Ground | 0 | 1 | 0 | 1 | 1 | 1 | 1 | 1 | 0 | 0 | 0 | 0 |
| *Carpodacus_pulcherrimus* | Granivores | Understorey | 0 | 0 | 0 | 0 | 0 | 1 | 1 | 1 | 0 | 0 | 0 | 0 |
| *Carpodacus_rubicilloides* | Granivores | Ground | 0 | 0 | 0 | 0 | 0 | 0 | 0 | 1 | 1 | 1 | 0 | 0 |
| *Carpodacus_rubicilla* | Granivores | Ground | 0 | 0 | 0 | 0 | 0 | 0 | 0 | 0 | 1 | 0 | 1 | 0 |
| *Carpodacus_edwardsii* | Granivores | Ground | 0 | 0 | 0 | 0 | 0 | 0 | 0 | 1 | 1 | 0 | 0 | 0 |
| *Carpodacus_erythrinus* | Granivores | Understorey | 0 | 0 | 0 | 0 | 0 | 1 | 0 | 1 | 1 | 1 | 0 | 0 |
| *Carpodacus_rodopeplus* | Granivores | Understorey | 0 | 0 | 0 | 0 | 0 | 1 | 1 | 0 | 0 | 0 | 0 | 0 |
| *Carpodacus_nipalensis* | Omnivores | Ground | 0 | 0 | 0 | 0 | 1 | 1 | 0 | 1 | 0 | 0 | 0 | 0 |
| *Leucosticte_brandti* | Granivores | Ground | 0 | 0 | 0 | 0 | 0 | 0 | 0 | 1 | 1 | 1 | 1 | 1 |
| *Pernis_ptilorhyncus* | Carnivores | Air | 0 | 0 | 1 | 1 | 1 | 1 | 0 | 0 | 0 | 0 | 0 | 0 |
| *Gypaetus_barbatus* | Carnivores | Air | 0 | 0 | 0 | 0 | 0 | 0 | 0 | 1 | 1 | 1 | 1 | 1 |
| *Gyps_himalayensis* | Carnivores | Air | 0 | 0 | 0 | 1 | 1 | 1 | 1 | 1 | 0 | 0 | 0 | 0 |
| *Gyps_fulvus* | Carnivores | Air | 0 | 0 | 0 | 0 | 0 | 0 | 0 | 1 | 0 | 0 | 0 | 0 |
| *Milvus_migrans* | Carnivores | Air | 0 | 0 | 0 | 0 | 0 | 1 | 0 | 0 | 0 | 0 | 0 | 0 |
| *Buteo_hemilasius* | Carnivores | Air | 0 | 0 | 0 | 0 | 0 | 0 | 1 | 0 | 0 | 1 | 0 | 0 |
| *Accipiter_badius* | Carnivores | Air | 0 | 0 | 1 | 1 | 0 | 0 | 0 | 0 | 0 | 0 | 0 | 0 |
| *Accipiter_trivirgatus* | Carnivores | Air | 1 | 0 | 0 | 0 | 0 | 0 | 0 | 0 | 0 | 0 | 0 | 0 |
| *Aquila_chrysaetos* | Carnivores | Air | 1 | 0 | 0 | 0 | 0 | 1 | 0 | 0 | 0 | 0 | 0 | 0 |
| *Hieraaetus_pennatus* | Carnivores | Air | 1 | 0 | 1 | 1 | 0 | 0 | 0 | 0 | 0 | 0 | 0 | 0 |
| *Upupa_epops* | Insectivores | Ground | 0 | 0 | 0 | 1 | 0 | 1 | 0 | 1 | 1 | 1 | 1 | 1 |
| *Megalaima_virens* | Granivores | Canopy | 1 | 1 | 1 | 0 | 0 | 0 | 0 | 0 | 0 | 0 | 0 | 0 |
| *Picus_squamatus* | Insectivores | Midstorey | 1 | 1 | 1 | 1 | 1 | 1 | 0 | 0 | 0 | 0 | 0 | 0 |
| *Dendrocopos_auriceps* | Insectivores | Midstorey | 0 | 1 | 0 | 0 | 0 | 0 | 0 | 0 | 0 | 0 | 0 | 0 |
| *Dendrocopos_cathpharius* | Insectivores | Midstorey | 0 | 1 | 0 | 0 | 0 | 0 | 0 | 0 | 0 | 0 | 0 | 0 |
| *Apus_affinis* | Insectivores | Air | 1 | 1 | 1 | 1 | 1 | 1 | 1 | 1 | 0 | 0 | 0 | 0 |

***Notes***: ele1, 1800-2100 m asl; ele2, 2100-2400 m asl; ele3, 2400-2700 m asl; ele4, 2700-3000 m asl; ele 5, 3000-3300 m asl; **ele6, 3300-3600 m asl**; ele7, 3600-3900 m asl; ele8, 3900-4200 m asl; ele9, 4200-4500 m asl; ele10, 4500-4800 m asl; ele11, 4800-5100 m asl; ele12, 5100-5400 m asl.

**Table S2 Richness of understorey, midstorey, canopy, air guilds in each elevation band across the entire elevation gradient**

| **Band** | **Elevation** | **Understorey** | **Midstorey** | **Canopy** | **Air** | **The combinations** |
| --- | --- | --- | --- | --- | --- | --- |
| ele1 | 1800-2100 | 5 | 4 | 17 | 4 | 30 |
| ele2 | 2100-2400 | 7 | 8 | 19 | 1 | 35 |
| ele3 | 2400-2700 | 12 | 10 | 25 | 4 | 51 |
| ele4 | 2700-3000 | 18 | 8 | 18 | 5 | 49 |
| ele5 | 3000-3300 | 17 | 9 | 14 | 4 | 44 |
| ele6 | 3300-3600 | 20 | 8 | 10 | 5 | 43 |
| ele7 | 3600-3900 | 20 | 5 | 10 | 4 | 39 |
| ele8 | 3900-4200 | 10 | 2 | 5 | 6 | 23 |
| ele9 | 4200-4500 | 5 | 1 | 0 | 4 | 10 |
| ele10 | 4500-4800 | 5 | 1 | 0 | 4 | 10 |
| ele11 | 4800-5100 | 2 | 1 | 0 | 2 | 5 |
| ele12 | 5100-5400 | 1 | 0 | 0 | 1 | 2 |

Table S3 Shapiro-Wilk normality tests for overall bird richness and guild richness

|  | W values | *P* values |
| --- | --- | --- |
| All birds | 0.92279 | 0.3099 |
| Granivores | 0.93453 | 0.4306 |
| Insectivores | 0.9075 | 0.1982 |
| Omnivores | 0.95535 | 0.7159 |
| Ground feeding birds | 0.94553 | 0.5728 |

**Table S4 Comparison between t**he model showing the highest explanatory power and lowest variance inflation factor (VIF) values (< 10) and the full model including seven explanatory variables.

|  | VIF values | | | | | | | AICc | *R*^2^ adj | *P* |
| --- | --- | --- | --- | --- | --- | --- | --- | --- | --- | --- |
|  | Area | MAP | PSR | HH | MAT | NDVI | MDE |  |  |  |
| All birds | 70.172 | 87.046 | 8.791 | 17.545 | 119.811 | 58.082 | 36.041 | 142.22 | 0.991 | <0.001 |
|  | / | / | 5.911 | / | / | 5.949 | 1.045 | 77.191 | 0.968 | <0.001 |
| Granivores | 66.328 | 102.572 | 8.842 | 19.266 | 119.109 | 64.123 | 40.308 | 143.34 | 0.798 | 0.057 |
|  | / | / | 5.911 | / | / | 5.944 | 1.04 | 65.144 | 0.746 | 0.004 |
| Insectivores | 75.315 | 72.749 | 8.436 | 16.82 | 122.623 | 49.956 | 33.405 | 149.202 | 0.962 | 0.002 |
|  | / | / | 5.91 | 1.019 | / | 5.931 | / | 72.443 | 0.947 | <0.001 |
| Omnivores | 66.187 | 103.119 | 9.403 | 17.524 | 116.266 | 69.345 | 37.806 | 140.61 | 0.803 | 0.054 |
|  | / | / | 5.91 | / | / | 5.956 | 1.046 | 54.668 | 0.87 | <0.001 |
| Ground feeding species | 80.059 | 73.369 | 8.754 | 16.108 | 129.871 | 51.041 | 33.731 | 121.183 | 0.99 | <0.001 |
|  | / | 3.755 | 3.799 | / | / | / | 1.385 | 85.549 | 0.552 | 0.037 |

**AICc, The corrected Akaike's Information Criterion. MAP, mean annual precipitation, PSR, plant species richness, HH, habitat heterogeneity, MAT, mean annual temperature, NDVI, the normalized difference vegetation index, and MDE, the mid-domain effect.**

**Table S5** Polynomial regression of the overall species richness patterns across the elevational bands.

| Regressions | All birds | Granivores | Insectivores | Omnivores | Ground feeding species |
| --- | --- | --- | --- | --- | --- |
| First-order *R*^2^ | 0.43* | 0.077 | 0.54** | 0.40 | 0.23 |
| AICc | 1791.2 | 139.81 | 592.52 | 76.65 | 353.25 |
| Second-order *R*^2^ | 0.92*** | **0.68**** | 0.88*** | **0.83***** | 0.55* |
| AICc | 249.08 | **56.18** | 160.37 | **28.76** | 213.18 |
| Third-order *R*^2^ | **0.94***** | 0.68* | **0.91***** | 0.87*** | **0.91***** |
| AICc | **203.07** | 60.49 | **123.99** | 29.50 | **54.19** |

*Significant at *P <* 0.05; **Significant at *P <* 0.01, ***Significant at *P <* 0.001. Bold entries indicate the best regression model selected by the lowest AICc values.

**Table S6 The relative importance and standard coefficient (shown in parentheses) of the variables included in the model-averaging analysis. The bold text indicates the most important variables (blue, positive values; red, negative values, the same below).**

|  | All birds | Granivores | Insectivores | Omnivores | Ground feeding species |
| --- | --- | --- | --- | --- | --- |
| Area | **0.41 (-0.48)** | 0.23 (-0.57) | 0.11 (-0.59) | 0.31 (0.81) | **1.00 (-2.19)** |
| MDE | **0.41 (0.55)** | **0.93 (1.31)** | 0.12 (0.28) | **0.55 (0.55)** | **1.00 (2.16)** |
| MAP | **0.59 (-0.70)** | 0.16 (-0.29) | 0.07 (-0.23) | 0.13 (-0.09) | **1.00 (-1.36)** |
| MAT | 0.04 (-0.17) | 0.14 (-0.15) | 0.09 (-0.49) | 0.09 (0.38) | <0.01 (2.70) |
| HH | **0.61 (0.47)** | 0.43 (-1.04) | **0.91 (0.47)** | 0.18 (0.41) | **1.00 (-0.92)** |
| PSR | 0.02 (-0.02) | 0.11 (-0.35) | 0.04 (0.09) | 0.34 (0.61) | <0.01 (-0.21) |
| NDVI | **0.99 (0.96)** | 0.21 (0.78) | **0.96 (0.84)** | **0.61 (1.14)** | **1.00 (-1.34)** |

**MDE, the mid-domain effect, MAP, mean annual precipitation, MAT, mean annual temperature, HH, habitat heterogeneity, PSR, plant species richness, and NDVI, the normalized difference vegetation index.**

Table S7 Significant interactions between guild identities and the various environmental/spatial factors.

| Coefficients: |  |  |  |  |
| --- | --- | --- | --- | --- |
|  | Estimate | Standard Error | t value | Pr (>|t|) |
| (Intercept) | 14.46 | 56.19 | 0.257 | 0.80 |
| Insectivores: Habitat heterogeneity | 27.89 | 7.55 | 3.692 | 0.002** |
| Ground feeding birds: NDVI | -48.59 | 22.73 | -2.138 | 0.048* |

**Fig. S1 The independent contribution (%) of each variable selected in hierarchical partitioning to the overall bird and guild richness. PSR, plant species richness, NDVI, the normalized difference vegetation index, MDE, the mid-domain effect, HH, habitat heterogeneity, and MAP, mean annual precipitation.**

SHAPE \* MERGEFORMAT

**Fig. S2. The phylogenetic tree of 151 species recorded in the central Himalaya, and the distribution of bird diet guilds (carnivores, granivores, insectivores and omnivores) in the tree. The phylogenetic tree was downloaded from a global phylogeny of birds through the website:** [**birdtree.org**](http://www.birdtree.org/)**.**
